# Sympathetic nerves are sparsely distributed in rat mesenteric perivascular adipose tissue

**DOI:** 10.3389/fphys.2025.1547785

**Published:** 2025-05-23

**Authors:** William F. Jackson, Emma D. Flood, D. Adam Lauver, Gregory D. Fink, Stephanie W. Watts, Brian D. Gulbransen

**Affiliations:** ^1^ Department of Pharmacology and Toxicology, College of Osteopathic Medicine, Michigan State University, East Lansing, MI, United States; ^2^ Department of Physiology, College of Natural Science, Michigan State University, East Lansing, MI, United States

**Keywords:** perivascular adipose tissue, sympathetic nerves, CD-31, mesenteric resistance arteries, rat

## Abstract

Perivascular adipose tissue (PVAT) importantly affects the contractile function of conduit and resistance arteries. Some findings suggest that this effect of PVAT is controlled in part by sympathetic neural input directly to various PVAT depots. However, the degree of innervation of PVAT by the sympathetic nervous system remains in question. Studies of murine mesenteric PVAT suggest limited innervation by tyrosine hydroxylase (TH)-positive nerves. The purpose of the present study was to extend these studies to rat mesenteric PVAT, particularly in Dahl-salt sensitive (Dahl-SS) rats, an important model of hypertension after high-fat diet consumption. Whole-mounts of mesenteric PVAT, mesenteric resistance arteries or small intestine were fixed, blocked with donkey serum and incubated with primary antibodies directed against rat TH (rabbit) and for CD-31 (PECAM, mouse monoclonal) as a control for antibody penetration. After washing and re-blocking with donkey serum, tissues were incubated with fluorescently labeled secondary antibodies. Z-stacks of the fluorescently labeled tissues were then acquired and fluorescence of labeled structure quantified in background-subtracted, maximum-intensity z-projections after thresholding. Transmitted light images were also captured to measure the width of PVAT surrounding mesenteric resistance arteries and PVAT adipocyte diameters. In PVAT from male Sprague-Dawley rats and in male and female Dahl-SS rats we found limited innervation by TH-positive nerves with most TH-positive nerves tracking along small blood vessels in the PVAT. In contrast, the expected strong labeling of TH-positive nerves on the surface of mesenteric resistance arteries was observed. The low level of TH-labeling in PVAT was not due to lack of antibody penetration or inability to image, because CD-31 labeled blood vessels were readily detected. Both TH and CD-31 labeling were also always detected in small intestine whole mounts. These data support the hypothesis that there is sparse innervation of mesenteric PVAT by TH-positive nerves and suggest limited direct control of individual adipocytes by sympathetic nerves.

## 1 Introduction

Perivascular adipose tissue (PVAT) refers to the fat tissue that surrounds many conduit and resistance arteries (Saxton et al., 2019a; Kim et al., 2020). Adipocytes are the most abundant cell-type in this tissue. Perivascular adipose tissue adipocytes influence vascular function in both health and disease by releasing vasoactive factors (adipokines) that blunt vasoconstriction in health but enhance vasoconstriction in disease states like obesity or hypertension (Saxton et al., 2019a).

The sympathetic nervous system plays a major role in controlling lipolysis in white adipose tissues other than PVAT by releasing catecholamines from the adrenal medulla and sympathetic nerve terminals (Correll, 1963; Bartness et al., 2014). These catecholamines are thought to drive the anti-contractile function of PVAT by engaging β_3_-adrenergic receptors and stimulating PVAT to release anti-contractile factors (Bussey et al., 2018; Saxton et al., 2018). Support for this concept was obtained from experiments showing that incubation of PVAT with tetrodotoxin (to inhibit fast sodium channel-mediated nerve action potentials) or 6-hydroxydopamine (to deplete norepinephrine from sympathetic nerve terminals) inhibited the electrical field stimulation-induced release of anti-contractile substances from PVAT (Saxton et al., 2018). These data were interpreted to imply that PVAT adipocytes are innervated by sympathetic nerves that stimulate the release of anticontractile substances from PVAT adipocytes.

In contrast, early studies in canine mesenteric adipose tissue (the PVAT around mesenteric resistance arteries) showed that nerve stimulation had little effect on lipolysis despite producing marked vasoconstriction demonstrating the efficacy of electrical stimulation (Ballard and Rosell, 1969). Only high concentrations of catecholamines administered intraarterially increased lipolysis from canine mesenteric PVAT (Ballard and Rosell, 1969). These data suggested that canine mesenteric PVAT adipocytes may not be controlled by sympathetic nerves and that control of mesenteric PVAT function by the sympathetic nervous system may differ from other white adipose tissue depots where neurally-released catecholamines appear to control lipolysis. Specifically, electrical stimulation of sympathetic nerves significantly increased lipolysis in rat and rabbit epidydimal adipose tissue (Correll, 1963), canine subcutaneous adipose tissue (Rosell, 1966; Fredholm and Rosell, 1968) and canine omental adipose tissue (Ballard and Rosell, 1971). Thus, there may be heterogeneity in sympathetic control of adipocyte function.

Catecholamines are present in PVAT but most of the catecholamines are located in the adipocytes (Ayala-Lopez et al., 2014; Ayala-Lopez et al., 2015; Ahmad et al., 2019). What remains unclear is the density of sympathetic innervation in PVAT with data supporting and refuting the hypothesis that adipose tissue is sparsely innervated by sympathetic nerves. Consistent with the sparse distribution hypothesis, Wirsen (1964) and Diculescu and Stoica (1970) fluorescently labeled catecholamines (Falck et al., 1962) to identify adrenergic nerve fibers in rat adipose tissue but found limited direct evidence of adipocyte innervation. Further evidence obtained using fluorescent labeling of catecholamines (Lindvall and Björklund, 1974) and electron-microscopic examination of rat mesenteric adipose tissue (PVAT around mesenteric resistance arteries) demonstrated plexi of adrenergic nerves on mesenteric resistance arteries, and along PVAT arterioles and venules with single fibers coursing next to capillaries (Slavin and Ballard, 1978). Occasional nerve fibers were observed adjacent to adipocytes. It was estimated that only 2%–3% of the adipocytes were innervated (defined as being within 40–200 nm of a nerve terminal) (Slavin and Ballard, 1978). A comparable low density of sympathetic innervation has been observed in murine mesenteric PVAT using immunofluorescent labeling of tyrosine hydroxylase (Murano et al., 2009) and in murine inguinal fat pads using genetically labeled TH-expression visualized by 2-photon microscopy (Zeng et al., 2015). A low density of sympathetic innervation of adipocytes in optically cleared murine epididymal adipose tissue, despite the typical dense innervation of arteries within the tissue, has been reported using three-dimensional 2-photon imaging of TH-positive nerves (Chi et al., 2018).

We have observed limited anatomical or functional evidence of adrenergic innervation of adipocytes in murine mesenteric PVAT through a series of experiments that incorporated genetic tracers, immunofluorescent labeling in optically cleared tissue, and adipocyte Ca^2+^ imaging in response to electrical nerve stimulation (Hanscom et al., 2024). These data support that hypothesis that there may be limited sympathetic innervation of adipocytes in mesenteric PVAT.

In contrast to studies suggesting a low density of sympathetic nerves in white adipose tissue, volume imaging of cleared murine inguinal adipose tissue suggested extensive potential innervation by TH-positive nerves (Jiang et al., 2017). These data were supported by electron microscopy evidence of nerve terminals with synaptic vesicles adjacent to adipocytes (Jiang et al., 2017) as reported previously (Slavin and Ballard, 1978). This is consistent with immunolabeling for dopamine-β-hydroxylase in sections of mouse mesenteric PVAT that appeared to show dense labeling of adrenergic nerves surrounding every adipocyte (Saxton et al., 2018), the lone study suggesting a high density of innervation of mesenteric PVAT. Similar evidence was obtained by three-dimensional 2-photon imaging of TH-positive nerves in cleared murine subcutaneous adipose tissue that identified a dense network of TH-labeled nerves in subcutaneous adipose tissue with the typical meshwork of innervation on larger blood vessels and with TH-positive neurons near most adipocytes (Chi et al., 2018). However, as noted previously, using the same approach in epididymal adipose tissue, despite the typical labeling of nerves on blood vessels, there were few TH-positive nerves near adipocytes (Chi et al., 2018). Thus, the density of innervation of adipocytes in all adipose tissue remains unclear with data suggesting that there may be heterogeneity of sympathetic innervation among adipose tissue depots.

Given the important functions of PVAT in vascular homeostasis (Saxton et al., 2019b) and the lack of consensus on sympathetic innervation density in mesenteric PVAT, the primary purpose of the present study was to use high resolution confocal microscopy and immunolabeling to test the hypothesis that PVAT around rat mesenteric resistance arteries is sparsely innervated by TH-positive sympathetic nerves. Obesity, such as that induced by a high fat diet, induces remodeling of adipose tissue (Sun et al., 2011) resulting in adipocyte hypertrophy and hyperplasia and a reduction in capillary density (Paavonsalo et al., 2020). High fat diet has previously been shown to modulate sympathetic neurotransmission in Dahl-SS rats in a sex-dependent manner (Alula et al., 2019). In addition, high fat feeding increases blood pressure in Dahl-SS rats (Alula et al., 2019; Fernandes et al., 2018; Watts et al., 2021). Therefore, our secondary goals were to assess the effects of high-fat feeding on PVAT sympathetic nerve innervation and to assess if there are sex-based differences in PVAT sympathetic nerve innervation in this model. We elected to study the innervation of rat mesenteric PVAT because PVAT innervation had not been previously studied in this species, and we used two strains of rat (see methods) to ensure that our results were not rat strain specific. Our data extend the idea that mesenteric PVAT is sparsely innervated by TH-positive sympathetic nerves from mice to rats and suggest that mostly the adipocytes that are immediately adjacent to innervated blood vessels are close to TH-positive sympathetic nerves. Our data also suggest that high-fat feeding or biological sex does not alter this relationship.

## 2 Methods

### 2.1 Animal models

All animal use followed IACUC regulations and approval at Michigan State University (Animal use protocol numbers: 10/14-193-00, 10/17-179-00 and PROTO202000009). Groups included male Sprague-Dawley (SD, Charles River strain #001) rats fed Control [n = 4, body weight ∼ 400 g as reported (Alula et al., 2019)] or High Fat Diets [n = 4, body weight ∼ 460 g as reported (Alula et al., 2019)] for 16–17 weeks, male Dahl salt-sensitive rats (Dahl-SS, Charles River strain #320) fed control diet (n = 6, body weight 427 ± 41, n = 5 as the weight of one rat was not recorded) and male and female Dahl-SS rats fed Control (n = 5) or High Fat (n = 5) diets for 16–17 weeks (see Results Section for body weights of Dahl-SS rats in this group). Control diet was 10% kCal fat (lard) and 0.2% Na (Diet D12450J, Research Diets, New Brunswick, NJ, United States) while the High Fat diet was 60% kCal high fat (lard) and 0.3% Na (Diet D12492). Rats were weighed and then they were euthanized with 5% isoflurane in 100% O_2_ followed by a bilateral pneumothorax. After shaving the abdomen, the entire mesentery was promptly removed and placed in phosphate buffered saline solution (PBS). A section from the middle of the small intestine was then isolated, pinned flat in a Sylgard-bottomed plastic Petri dish and rinsed 3X with PBS prior to macro-imaging and processing for immunofluorescent labeling of tyrosine hydroxylase to label sympathetic nerve fibers and CD-31 to label blood vessels that perfuse PVAT.

### 2.2 Immunolabeling

Preliminary experiments suggested sparse labeling of TH-positive structures resembling nerves in Sprague-Dawley mesenteric PVAT. Therefore, as a positive control for antibody penetration and labeled-structure visibility, we also used antibodies to label blood vessels in the PVAT microcirculation and mesenteric resistance arteries which are highly innervated by sympathetic nerves (Looft-Wilson and Gisolfi, 2002; Mui et al., 2018). Small sections (∼25–100 mm^2^) of Sprague-Dawley mesentery containing a mesenteric resistance artery surrounded by PVAT, or dissected lengths (5 mm) of mesenteric resistance arteries were removed and pinned flat in Sylgard-containing 35 mm Petri dishes. Sprague-Dawley tissues were permeabilized with PBS containing 0.1% saponin and 0.05% sodium azide (Saponin PBS), blocked for 120 min with 4% donkey serum in Saponin PBS and then incubated with mouse anti-rat CD-31 primary antibodies (Mouse monoclonal TLD-3A12, Invitrogen MA1-80069; RRID#: AB_928130; 1:200) overnight. After washing 5X with Saponin PBS and blocking with 4% donkey serum in saponin PBS, tissues were incubated with AlexaFluor 594-labeled donkey-anti-mouse secondary antibodies (Invitrogen A32744; RRID#: AB_2762826; 1:100) for 2 h. This pre-fixative staining was required because CD-31 antibodies would not reliably label vessels in Sprague-Dawley mesenteric PVAT after paraformaldehyde fixation. The tissues were then rinsed 5X with saponin-PBS and fixed for 30 min with freshly made 4% paraformaldehyde. The tissues then were rinsed 5X with PBS, permeabilized with Saponin PBS, blocked with donkey-serum in PBS (120 min) and labeled with rabbit-anti-rat tyrosine hydroxylase (OPA1-04050, Invitrogen; RRID#: AB_325653; 1:500) overnight. After 5X wash with saponin PBS and blockade with donkey-serum in saponin solution, tissues were exposed to AlexaFluor 488-labeled, donkey-anti-rabbit secondary antibodies (Invitrogen A-21206; RRID#: AB_2535792; 1:100) for 2 h. A 5X rinse with saponin PBS was then followed by 5X rinse with PBS containing 0.05% sodium azide and the tissues were placed on a clean glass slide with 1-2 drops Vectashield (H-1000, Vector) mounting medium and covered with a CoverWell Imaging Chamber (#70327-10, Electron Microscopy, Sciences). As a control for non-specific labeling, primary antibodies were omitted from some tissues.

We attempted a similar approach with tissues from male Dahl-SS rats. However, the anti-CD-31 antibodies that worked well in Sprague-Dawley tissues would not reliably label vessels in unfixed or paraformaldehyde-fixed Dahl-SS PVAT. However, there appeared to be specific labeling of nerves in paraformaldehyde-fixed Dahl-SS tissue, as will be shown in the Results section, using the approach described above for TH-staining in Sprague-Dawley PVAT.

Dahl-SS rat tissue was alternatively fixed with an ethanol-based fixative [ethanol 70%, glycerol 1%, glacial acetic acid 0.5%, 0.5 PBS 28.5%; (Chung et al., 2018)] and labeled for both TH and CD-31 in the same tissue. Sections of intestine with attached mesenteries were fixed overnight in the ethanol-based fixative and then rinsed briefly with PBS. Quadruple 25–100 mm^2^ samples of intestine (to serve as a positive control for both TH- and CD-31-staining), mesenteric resistance arteries surrounded by PVAT, and similar samples in which we surgically exposed the surface of the mesenteric resistance arteries (positive control for TH-staining) were dissected and pinned to the bottom of Sylgard-lined 48 well culture dishes. This allowed, for each animal and tissue, a negative control (no antibody exposure), a secondary antibody-only control, and duplicate samples exposed to both primary and secondary antibodies. Samples were washed 3X with PBS, then permeabilized with PBS containing 0.1% Triton X-100 and 0.05% sodium azide (Triton X-100 PBS, 3X). The tissues then were blocked with Triton X-100 PBS containing 10% donkey serum for 1 h before incubating in primary antibodies (mouse anti-rat CD31 monoclonal antibody TDL-3A12 Invitrogen MA5-16951 1:200 and Rabbit anti-rat TH tyrosine hydroxylase polyclonal antibody Invitrogen OAP1-04050 1:200) or vehicle (Triton X-100 PBS containing 5% donkey Serum) overnight at 4°C. The next day, samples were washed 3X and blocked again with 10% donkey Serum in Triton X-100 PBS before incubating with secondary antibodies (donkey anti rabbit AlexaFluor 488-labeled secondary antibody, Invitrogen A21206 and donkey anti mouse AlexaFluor 594-labeled secondary antibody, Invitrogen 1:100) or vehicle (Triton X-100 PBS containing 5% donkey Serum) for 2 h. Tissues then were washed 3X in Triton X-100 PBS and 3X with PBS containing 0.05% sodium azide. Samples then were removed from the culture dish, mounted on slides with 1-2 drops of Vectashield (H-1000, Vector) mounting media and sealed with a CoverWell Imaging Chamber.

### 2.3 Image capture

**FIGURE 1:**
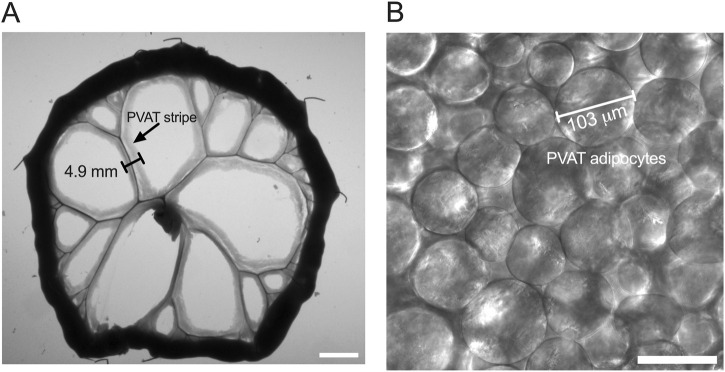
Measurement of PVAT stripe width and PVAT adipocyte diameter. Panel **(A)** shows an image of a section of small intestine from a male Sprague-Dawley rat on Control diet that has been pinned to a Sylgard pad and imaged as described in the text. The scale bar in Panel A = 10 mm. PVAT stripe width around second and third order mesenteric resistance arteries was measured as indicated in the figure. Panel **(** **B** **)** shows a transmitted light image of mesenteric PVAT that was used to measure PVAT adipocyte diameter as indicated in the figure. The scale bar in Panel B = 100 μm.

Macro-images of the pinned-out intestine, mesentery, the associated mesenteric resistance arteries and their PVAT were acquired prior to fixation using either a DMK33UX174 or a DFK21BU04.H CCD camera (Imaging Source) coupled to a Computar Macro 10 lens (Edmund Optics) (Figure 1A). These images were used to assess the effects of high-fat feeding on the width of the PVAT “stripe” surrounding mesenteric resistance arteries (Figure 1A). Widths were measured with FIJI version of ImageJ (Schindelin et al., 2012) using a mm ruler to calibrate the images with ∼10 sites per animal measured. The means of measurements from each rat were used as representative measures for statistical analysis.

Fluorescently-labeled tissue from Sprague-Dawley rats and paraformaldehyde-fixed Dahl-SS rats were imaged on a Nikon Eclipse TE2000-U inverted microscope equipped with 20X (0.5 NA) and 40X (0.75 NA) objectives, motorized focus, X-Cite metal halide illumination system and a Photometrics CoolSNAP ES cooled 12-bit high-resolution CCD camera controlled by MetaMorph software. Camera gain and illumination intensity were adjusted to maximize fluorescence emission intensity in tissues exposed to primary and secondary antibodies without significant autofluorescence in untreated or secondary only treated control tissues. Image stacks (∼50, 1392 X 1040-pixel 12bit images, 2 μm step size) were acquired with each channel (Alexa Fluor 488, Alexa Fluor 594, transmitted light) imaged sequentially to minimize overlap, bleed-through and other artifacts. Image stacks were saved as 16-bit .TIFF files for subsequent processing in FIJI version of ImageJ.

Fluorescently labeled tissue from Dahl-SS rats fixed with an ethanol-based fixative were imaged on a Zeiss LSM 880 NLO system equipped with 594 and 488 nm laser lines. Images were acquired using a Plan-Apochromat ×20 objective (NA = 0.8). Settings (photomultiplier gain, laser intensity, pin hole size) were optimized over a range of positive samples (i.e., those that were exposed to both primary and secondary antibodies), across all three tissue types (intestine, exposed mesenteric resistance artery, and PVAT) such that the fluorescence emission did not saturate the detectors, or create autofluorescence in the negative control samples (i.e., either those samples with no secondary antibody or samples that were not antibody treated). Once optimized, the same settings were used across all groups: male, female, high fat diet, or control diet. Image stacks (∼100, 3-channel, 1024X1024 8-bit images, 1 μm step-size) were acquired with sequential line scanning to reduce channel crosstalk. Images were saved in .czi file format for subsequent processing in FIJI version of ImageJ. Six randomly selected sites were imaged in each sample that was exposed to both primary and secondary antibodies, whereas only two randomly selected sites were imaged in tissues exposed to secondary only and one site imaged in samples not exposed to antibodies as these were consistently without fluorescent signal under the imaging conditions that were used. In addition to fluorescence, transmitted light images were acquired so that adipocyte diameters could be measured and so that the localization of immunostaining could be assessed.

### 2.4 Image analysis

Images were processed using FIJI version of ImageJ (Schindelin et al., 2012). Files were opened as a hyperstack, and the channels separated so that they could be analyzed separately. Adipocyte diameters were measured from the transmitted light images by selecting the images with in-focus adipocytes and measuring the maximum diameter of those cells (Figure 1B). We measured the diameters of ∼25 adipocytes per sample, and these diameters were averaged to obtain a representative sample for each animal for statistical analysis.

**FIGURE 2:**
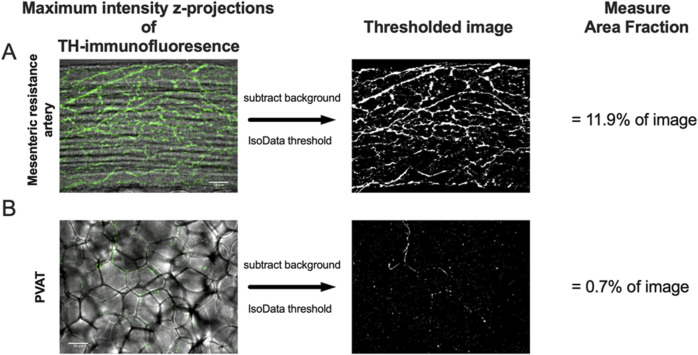
Image analysis protocol. Panels A and B show maximum-intensity z-projections of TH-immunofluorescence (green) of image stacks from the surface of a mesenteric resistance artery **(A)** and PVAT **(B)** as indicated from a male Sprague-Dawley rat on control diet. The z-projections are shown overlaid over transmitted light images of the artery and PVAT for reference. After background subtraction, the images were converted to binary (black and white) using the IsoData threshold in ImageJ and the Area Fraction (% of area occupied by white pixels) computed as an index of TH-positive nerve density. The scale bars (50 μm) shown in the left images in Panels A and B also applies to the binary images. See text for additional information.

Maximum intensity z-projections were computed to reduce dimensionality of the fluorescence image stacks. For intestine and PVAT samples, the full z stack was used in the projection. Only the image slices containing the upper wall of exposed mesenteric arteries was used in their z-projections. Background was subtracted using a 25-pixel sliding parabola in FIJI. Because we observed significant speckle-type background fluorescence in the ethanol-fixed Dahl-SS tissue, we applied a median filter with a pixel radius 1-3 to help reduce this non-nerve/vessel-related fluorescence. The z-projections then were converted to binary (black and white) images by applying the IsoData threshold with the lower limit adjusted as needed to limit background fluorescence. Figures 2A,B show representative z-projections before and after this image processing sequence. We recorded the threshold values of the samples exposed to both primary and secondary antibodies and used the mean of these values for thresholding the paired samples not exposed to primary antibodies. We then measured the fraction of image area occupied by white pixels as an index of antibody staining. Weaknesses of this approach are that all signal within the thresholding window will be counted including any background staining, and structures with larger dimensions will contribute more to the fraction of area than structures with smaller dimensions such that you cannot compare, for example, CD-31 staining (a measure of microvascular density) with TH-staining (presumably sympathetic nerves) because the microvessels have a larger diameter than the small nerve fibers that we observed. Nonetheless, this approach provides an index that reflects a reasonable estimate of the presence and density of labeled structures in samples.

Background, non-linear fluorescence was minimal in the paraformaldehyde-fixed tissues from Sprague-Dawley and Dahl-SS rats, while it was significant on some of the ethanol-fixed Dahl-SS tissues. Therefore, as an alternative, we also used the NeuronJ plug-in for FIJI on the ethanol-fixed Dahl-SS tissues that quantified the length of labeled structures in the z-projections that was independent from non-linear signals and independent of the radial dimensions of the labeled structures. As will be seen in the Results Section, comparable results were obtained with both methods of quantification.

### 2.5 Statistics

Data are presented as means ± SE with n-values representing the number of rats studied for each group. For all ANOVA designs, the assumption of homogeneity of variance was tested using the Brown-Forsythe test. If significant heterogeneity of variance was detected (p < 0.05), the data were transformed as needed (see results) to resolve this issue, and all statistical comparisons were performed on the transformed data. Two-way ANOVA was used to assess the effects of diet and biological sex on both the width of mesenteric PVAT stripes and the diameter of PVAT adipocytes with Fisher’s Least Significant Difference test used to compare means if the ANOVA was significant. Three-way ANOVA with primary antibody (with vs. without), diet (control vs. high fat), and biological sex (male vs. female) as the main factors was used to analyze the outcome of labeling for TH or CD-31 for each tissue studied (PVAT, mesenteric resistance artery or intestine). Šídák’s multiple comparisons test was used to compare means if the ANOVA indicated significant differences. GraphPad Prism 10.3 (GraphPad Software, LLC) was used for all statistics. P-values less than 0.05 were considered statistically significant.

## 3 Results

### 3.1 High fat feeding increases PVAT dimensions in male Sprague-Dawley Rats

**FIGURE 3:**
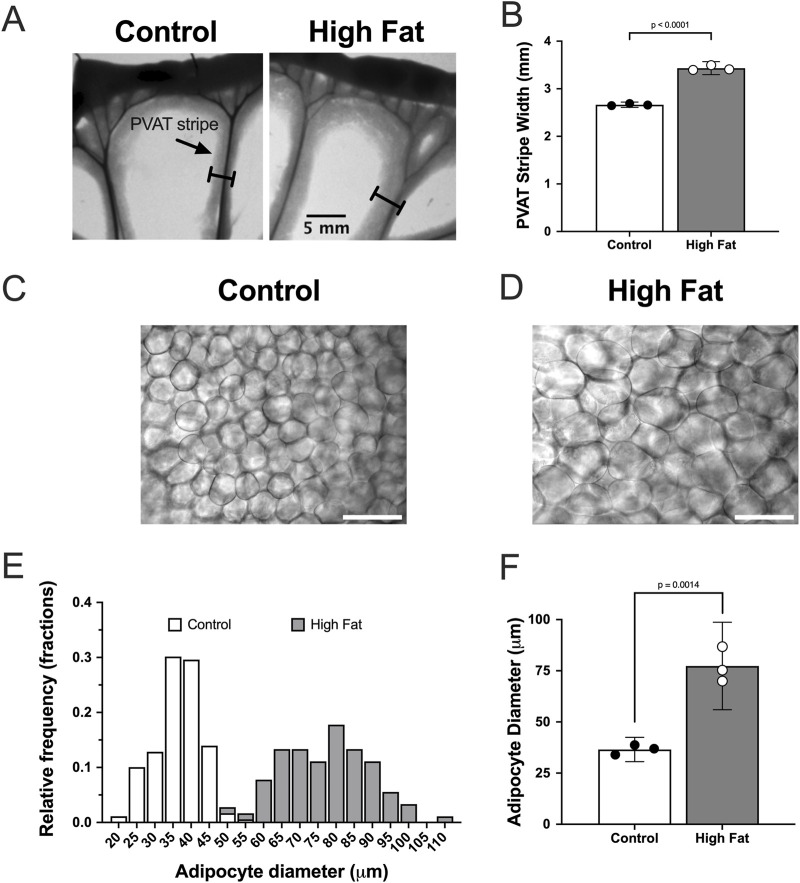
High fat feeding increases the size of mesenteric PVAT adipocytes in male Sprague-Dawley Rats. Panel **(A)** show images of mesenteric PVAT stripes around resistance arteries and veins removed from a control fed and high fat fed rat after 16 weeks on diet as indicated. The scale bar in the right image applies to both images. Panel **(B)** shows means ± SE (n = 3 rats per group) PVAT stripe width (p-value from, unpaired t-test). Panels **(C,D)** show transmitted light images of PVAT adipocytes from control and high-fat-fed rats as indicated. The scale bars in Panels **(C,D)** are 100 μm. Panel **(E)** shows the distribution of adipocyte diameters for control and high-fat fed animals. Panel **(F)** shows summary means ± SE (n = 3 rats per group) for adipocyte diameters (p-value from unpaired t-test).

High-fat-feeding for 16–17 weeks significantly increased the width of PVAT stripes surrounding mesenteric resistance arteries (an index of adiposity) in mesenteries from male Sprague-Dawley rats (Figures 3A,B). In addition, high-fat-feeding significantly increased the diameter of PVAT adipocytes (Figures 3C–F).

### 3.2 Mesenteric resistance artery PVAT is sparsely innervated by TH-positive nerves in male Sprague-Dawley rats

**FIGURE 4:**
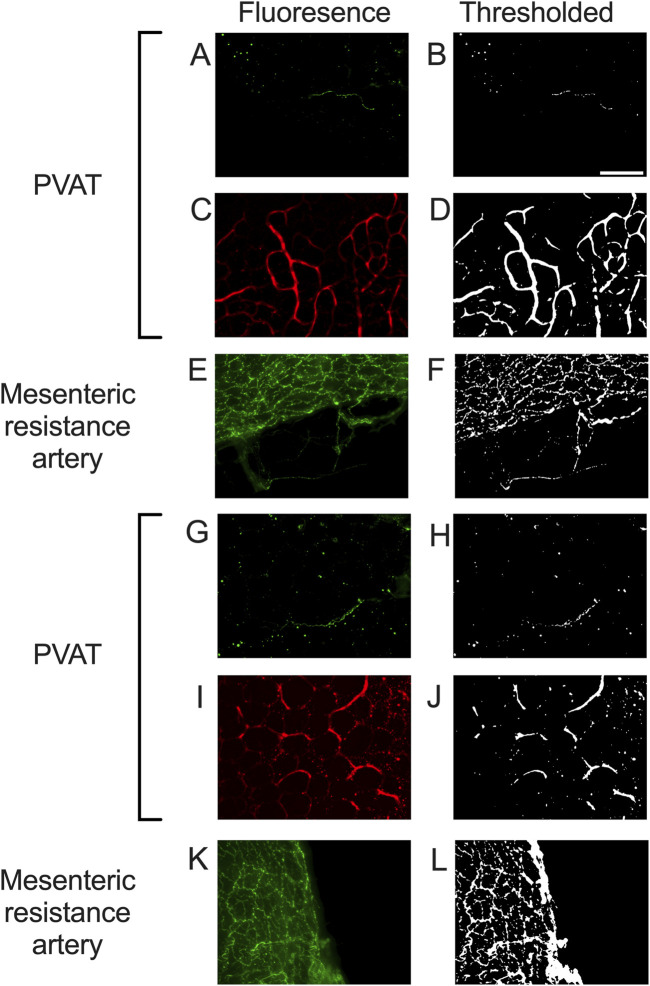
Representative images of tyrosine hydroxylase- and CD-31-labeling in PVAT and tyrosine hydroxylase on mesenteric resistance arteries of male Sprague-Dawley Rats. Panels **(A,C,E,G,I,K)** show fluorescence images of PVAT **(A,C,G,I)** or mesenteric resistance arteries **(E,K)** stained for tyrosine hydroxylase (**(A,E,G,K)**– green fluorescence) or CD-31 (**(C,I)** – red fluorescence) in a sample from control fed **(A,C,E)** or high fat fed **(G,I,K)** rats. Images in **(A,C,E,G,I,K)** are maximum intensity z-projections of z-stacks. Brightness and contrast were adjusted for display purposes only. Panels **(B,D,F,H,J,L)** show the corresponding thresholded images after background subtraction and median filtering that were used for quantification of the labeling density. Panels **(A,B,G,H)** show the typical low level of TH-staining observed in PVAT, Whereas TH was readily detected in mesenteric resistance arteries **(E,F,K,L)** and CD-31 was easily detected in PVAT **(C,D,I,J)**. The scale bar in Panel B is 100 μm and applies to all panels in the figure.

**FIGURE 5:**
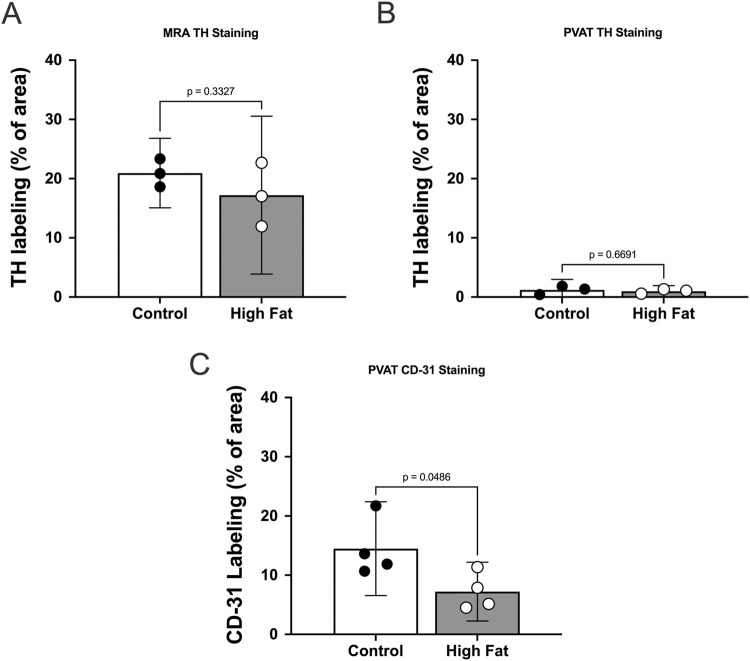
TH-immunofluorescence is sparse in mesenteric PVAT in male Sprague Dawley Rats and high fat feeding decreases CD-31 labeling in PVAT. Panels A–C show summary means ± SE (n = 3 rats per group) % of image area occupied by white pixels for mesenteric resistance arteries **(A)** and mesenteric PVAT **(B,C)** labeled for TH **(A,B)** or CD-31 **(C)** for control and high fat-fed rats as indicated. P-values from unpaired t-tests.

**FIGURE 6:**
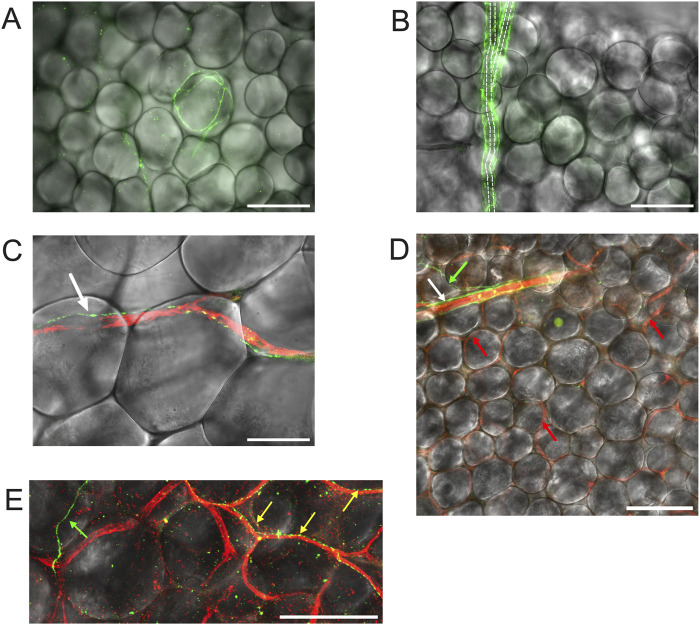
TH-labeling in mesenteric PVAT. Panel **(A)** shows mesenteric PVAT TH immunofluorescence (green) from a male Dahl-SS rat (paraformaldehyde-fixed tissue) overlaid on a transmitted light image of PVAT showing an example of the occasional adipocyte associated with TH-positive nerves. Panel **(B)** is also from a male Dahl-SS rat (paraformaldehyde-fixed tissue) showing TH-Positive nerves (green) encompassing a small arteriole. Dashed lines show the approximate location of the arteriole that branches at about its midpoint. Note that there were no other TH-labeled structures associated with the other adipocytes shown in the image. Panel C shows an example of a TH-positive nerve fiber (green) tracking a CD-31 positive blood vessel (red) in PVAT from a male Sprague-Dawley rat (paraformaldehyde-fixed tissue). The white arrow in Panel **(C)** points to the TH-positive nerve (green). Panel **(D)** shows an image from a female Dahl-SS rat (ethanol-based fixation) showing a small arteriole labeled for CD-31 (red) with associated TH-positive nerves (green) noted by the white arrow. The green arrow points to a TH-positive nerve (green) that appeared to branch from the nerves associated with the arteriole. The red arrows point to several of the CD-31-stained capillaries (red) that were labeled in this image. Note that other than the nerve indicated by the white arrow at the top of the image, no other TH-positive structures were associated with the rest of the adipocytes shown in the image. Panel **(E)** shows a zoomed imaged of TH-stained nerves (green and yellow) and CD-31-stained blood vessels in PVAT from a control diet-fed male Dahl-SS rat. The green arrow points to a TH-positive nerve passing over adipocytes and not associated with blood vessels. The yellow arrows point to TH-positive nerves that are tracking CD-31-positive blood vessels. The scale bars in panels **(A,B,D,E)** are 100 μm. The scale bar in panel C is 50 μm.

**FIGURE 7:**
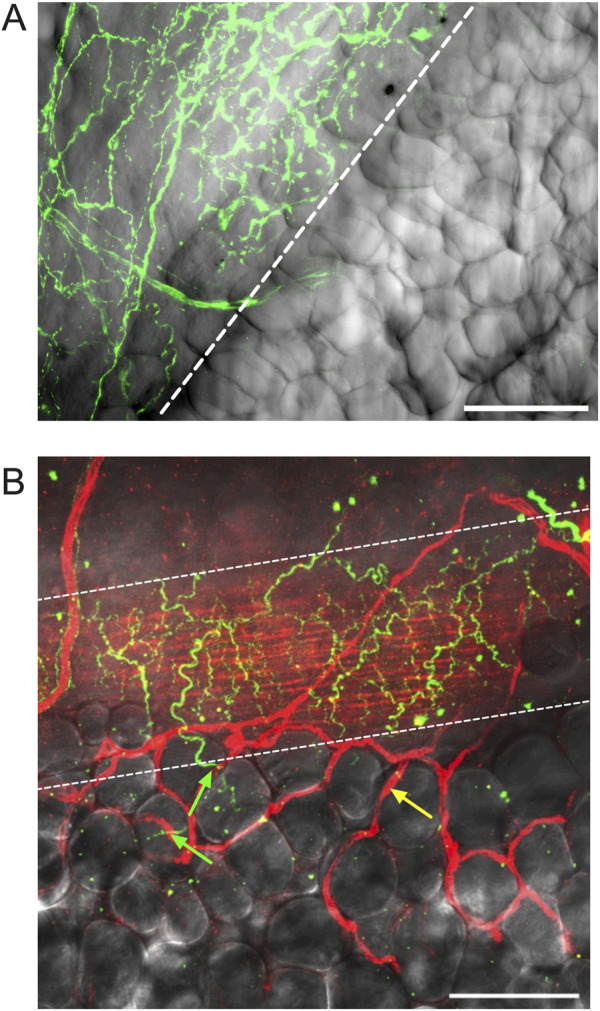
Adipocytes adjacent to mesenteric resistance arteries are near high densities of TH-positive nerves. Panel **(A)** is a z-projection of TH-immunofluorescence (green) on the surface of a mesenteric resistance artery from a male Dahl-SS rat that was paraformaldehyde-fixed and overlaid on transmitted light image of the vessel and adjacent PVAT. The first row of adipocytes overlies the nerves on the surface of the artery, whereas adipocytes farther away from the vessel do not appear to be associated with TH-immunofluorescence. Panel **(B)** is a similar image of the surface of a mesenteric resistance artery (ethanol-based fixation) from a female Dahl-SS rat fed a high-fat diet showing TH labeling on the vessel surface (green fluorescence) and CD-31-labeled endothelial cells (red labeling under the TH-positive nerves in green) as well as microvessels on the surface of the vessel and out in the adjacent PVAT (highlighted by yellow arrow). The green arrows point to TH-positive nerves that pass from the mesenteric resistance artery and appear associated with a few adipocytes. The yellow arrow points to a CD-31-positive blood vessel not associated with TH-staining. The scale bars in Panels A and B are 100 μm.

We could readily detect TH-positive nerves on the surface of mesenteric resistance arteries (Figures 2A, 4, 5A). However, as shown in Figures 4A,B,G,H, 5B there were very few TH-labeled structures in the PVAT surrounding mesenteric resistance arteries in male Sprague-Dawley rats fed Control or High-Fat diets. Similar results were observed in paraformaldehyde-fixed tissue from male Dahl-SS rats fed the control diet: 10% ± 1.6% (n = 5) of images were occupied by TH-positive structures on the surface mesenteric resistance arteries, whereas only 0.5% ± 0.07% (n = 5) of image area was occupied by TH-positive structures in the surrounding PVAT. In addition, as shown in Figures 4C,D,I,J, 5C, CD-31-positive blood vessels were always observed in PVAT samples demonstrating access of antibodies to structures around the adipocytes. Occasionally, we observed isolated adipocytes with TH labeled structures as shown in Figure 6A. However, TH-labeled structures were more often observed coursing along blood vessels as shown in Figure 6 in both Dahl-SS rat PVAT (Figures 6B,D) and in Sprague-Dawley rat (Figure 6C) PVAT. Also, as shown in Figure 7, while TH-immunofluorescence could be readily detected on the surface of mesenteric resistance arteries, adjacent PVAT often appeared to be mostly devoid of TH-positive structures, with only the layer of adipocytes immediately adjacent to the resistance artery being close to TH-positive nerves.

High-fat-feeding appeared to have no significant effect on TH-labeling on the surface of the mesenteric resistance arteries (Figure 5A) or within PVAT as shown in Figure 5B. In contrast, CD-31 labeling in PVAT of high-fat-fed Sprague-Dawley rats was about 50% of that in control-fed rats (Figure 5C). This is likely the result of the near doubling of the diameter of adipocytes in the high-fat-fed animals (Figure 3F).

### 3.3 High fat feeding has limited effect on mesenteric PVAT dimensions in Dahl-SS rats

**FIGURE 8:**
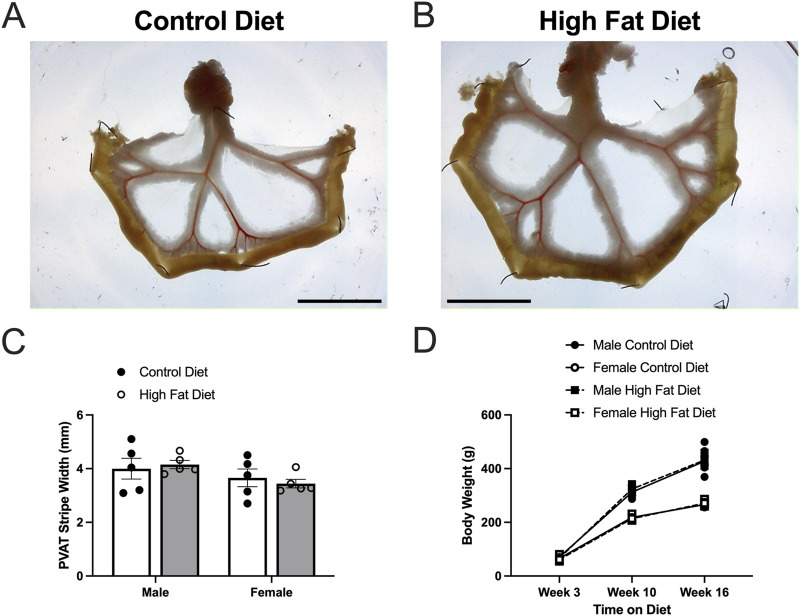
High fat diet did not increase body weight or PVAT stripe width in Dahl-SS rats. Panels **(A,B)** show representative images of mesenteric PVAT in control and high-fat-fed Dahl-SS rats. The scale bar in each image is 2 cm. Panel **(C)** shows summary mean ± SE PVAT stripe widths for male and female, control and high-fed Dahl-SS rats (n = 5). There was a tendency for males to have larger PVAT stripes than females, but this did not attain statistical significance by two-way ANOVA (p = 0.0767 for Sex, p = 0.9106 for Diet and p = 0.5187 for Sex X Diet interaction). Panel **(D)** shows mean ± SE body weights for male and female, control and high-fed Dahl-SS rats (n = 5). Brown-Forsythe test revealed significant heterogeneity of variance (p = 0.0111). Log10 transform of the data correct this issue (Brown-Forsythe test p = 0.2176). Three-way analysis of variance with repeated measures in the time on diet factor on the log10-transformed data revealed a significant effect of time on diet (p < 0.0001), sex (p < 0.0001) and a significant time on diet X sex interaction (p < 0.0001). Diet (p = 0.6978), time on diet X diet interaction (p = 0.2749), diet X sex interaction and time on diet X diet X sex interaction (p = 0.7411) were not significant. Males weighed more than females at both 10 and 16 weeks on diet.

**FIGURE 9:**
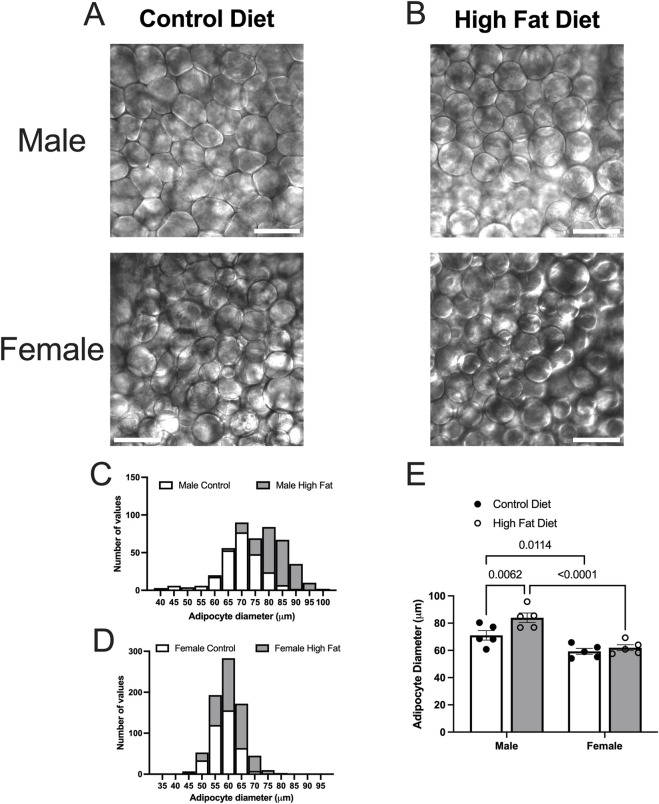
High fat diet increases adipocyte diameter in male Dahl-SS rats. Panels **(A,B)** show representative transmitted light images of mesenteric PVAT from male and female Dahl-SS rats fed with control **(A)** or high-fat **(B)** diets as indicated (scale bars = 100 μm). Panels **(C,D)** show the distributions of adipocyte diameters for all data. Panel E shows summary mean ± SE adipocyte diameters for male and female, control and high fat-fed Dahl-SS rats (n = 5). Two-way ANOVA indicated significant Sex (p < 0.0001) and Diet (p = 0.0163) effects with no significant Sex X Diet interaction (p = 0.0969). The high fat diet significantly increased adipocyte diameter only in males and the diameter of adipocytes was significantly greater in males compared to females as indicated. P-values shown are from Fisher’s LSD after 2-way ANOVA.

High-fat-feeding for 16–17 weeks appeared to have no significant effect on PVAT stripe width (Figures 8A–C) or body weight (Figure 8D) in this cohort of Dahl-SS rats. Males were heavier than females after 10 and 16 weeks on the high fat diet (Figure 8D). While there was a trend for PVAT stripe-width to be larger in males on a high fat diet, this did not attain statistical significance (p = 0.7046). High-fat-feeding did significantly increase PVAT adipocyte diameter in male Dahl-SS rats (Figures 9A–C,E), whereas mesenteric PVAT adipocytes in female Dahl-SS rats showed no significant hypertrophy (Figures 9D,E). In addition, adipocyte diameter was smaller in female compared to male Dahl-SS rats regardless of diet (Figure 9E).

### 3.4 Use of an ethanol-based fixative allowed both TH and CD-31 immunodetection in Dahl-SS PVAT

**FIGURE 10:**
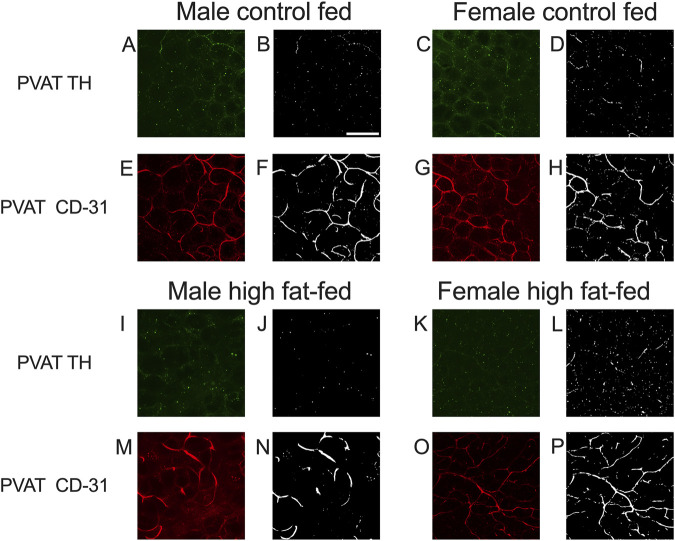
Representative images for TH and CD-31 staining in mesenteric PVAT from Dahl-SS rats. Images **(A,C,E,G,I,K,M,O)** are maximum intensity z-projections of z-stacks with contrast and brightness adjusted for display purposes only. Images **(B,D,F,H,J,L,N,P)** are the thresholded images used for quantification of label density after background subtraction and median filtering. Panels **(A,C,I,K)** show the typical low level of TH labeling (green fluorescence) in rat mesenteric PVAT from males and females fed either control or high fat diets as indicated. Images **(E,G,M,O)** show typical CD-31 labeling (red fluorescence) in the same image stack as for the TH images. Scale bar in Panel B is 100 μm and applies to all images in this figure.

**FIGURE 11:**
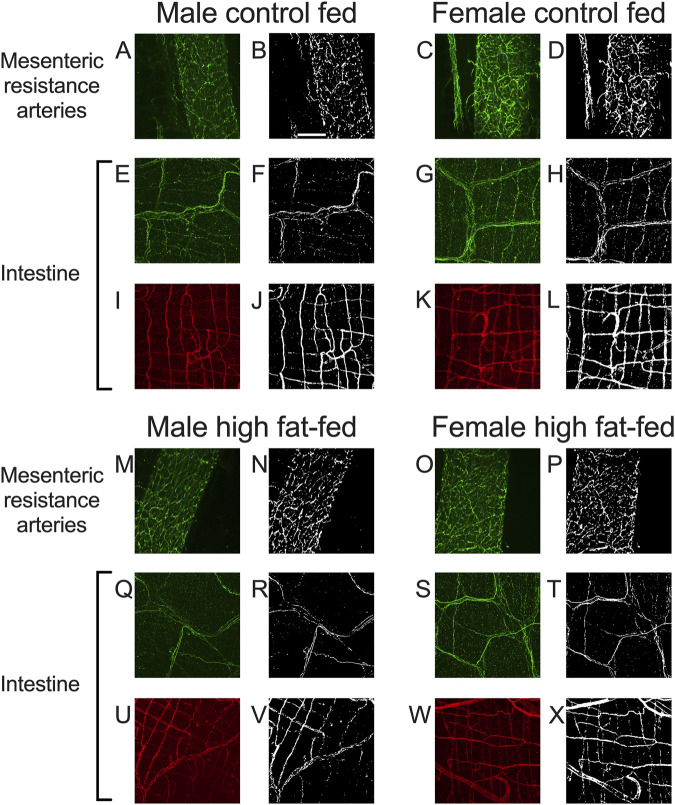
Representative labeling of TH in mesenteric resistance arteries and TH and CD-31 in small intestine of Dahl-SS rats. Images **(A,C,E,G,I,K,M,O,Q,S,U,W)** are maximum intensity z-projections of z-stacks with brightness and contrast adjusted for display purposes only. Images **(B,D,F,H,J,L,N,P,R,T,V,X)** are thresholded images used for quantification of labeling density after background subtraction and median filtering. Images **(A–D)** and **(M–P)** are TH labeling in mesenteric resistance arteries. Images **(E–H)** and **(Q–T)** are TH labeling in small intestine samples and images **(I–L)** and **(U–X)** are CD-31 labeling in the same small intestine samples as for TH. The scale bar in Panel **(B)** is 100 μm and applies to all images in this figure. These data show that TH-labeling and CD-31 labeling were readily detected in tissues from males and females fed control or high-fat diets as indicated in the figure.

As noted in the Methods Section, vascular endothelial cells in PVAT from Dahl-SS rats could not be reliably labeled in freshly harvested tissue or tissue fixed with paraformaldehyde. However, an ethanol-based fixative (Chung et al., 2018) allowed for immunolabeling of CD-31 and TH in the sample tissues. Figure 10 shows examples of labeling of both TH and CD-31 in samples of PVAT from male and female Dahl-SS rats on control or high-fat diet. As in tissues from male Sprague-Dawley or male Dahl-SS rats fixed with paraformaldehyde, limited TH-labeling was observed in PVAT whereas CD-31 labeling was readily observed. In contrast, TH labeling was always detected on the surface of mesenteric resistance arteries and both TH and CD-31 were labeled in whole-mounts of small intestine from Dahl-SS rats (Figure 11).

### 3.5 Mesenteric resistance artery PVAT is sparsely innervated by TH-positive nerves in Dahl-SS rats

**FIGURE 12:**
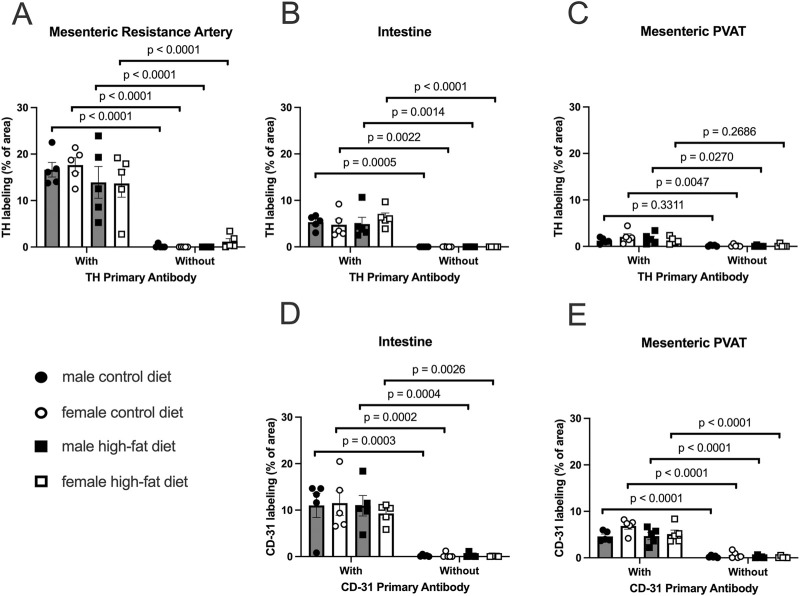
TH-positive nerves are sparsely distributed in Dahl SS mesenteric PVAT when analyzed as % of image area. Data are means ± SE (n = 5) % of image area occupied by labeled structures for TH (Panels A–C) for the surface of mesenteric resistance arteries **(A)**, whole-mounts of small intestine **(B)** and mesenteric PVAT **(C)** as indicated and for CD-31 for intestines **(D)** and mesenteric PVAT **(E)** for samples from both male and female rats fed control or high-fat diets. The legend shown in bottom left Panel applies to all other panels in the figure. Data were statistically analyzed by 3-way ANOVA with primary antibody (with or without), sex (male or female) and diet (control or high fat) as the main treatments. For TH-staining for all tissues (Panels A–C) and for both TH and CD-31 (Panels E and F), the ANOVAs indicated significant Antibody effects (p < 0.0001) with no significant sex or diet effects and no significant interactions (p > 0.05 – see Table 1 for exact p-values). The p-values shown in the figure refer to comparisons between means using Śídák’s multiple comparison test after a significant (p < 0.05) ANOVA result. The group variances for the data shown in Panel A were significantly different by the Brown-Forsythe test (p = 0.0097). Square-root transformation eliminated this issue (Brown-Forsythe p-value after transformation = 0.1043) and the p-values shown in Panel A were computed on the square-root transformed data.

In both mesenteric resistance arteries (Figures 11A–D,11M–P, 12A) and small intestine (Figures 11E–H,Q–T, 12B), our positive controls, significant TH-labeling was always observed (Figures 12A,B). In contrast and similar to our observations in paraformaldehyde-fixed tissue from Sprague-Dawley and male Dahl-SS rats, very limited TH-positive staining was observed in PVAT; only PVAT from female control-fed and male high-fat-fed being low, but significantly higher than the signal obtained from secondary antibody-only treated tissue (Figure 12C). In addition, as in our positive control (small intestine, Figures 11I–L,U–X, 12D) CD-31 immunofluorescence was always observed in PVAT from all groups (Figure 12E) suggesting that a lack of antibody access to structures outside of the adipocytes cannot explain the low level of TH staining observed in PVAT.

**TABLE 1:** 3-way ANOVA p-values for Figure 12.

Tissue	Antibody target	Primary antibody	Sex	Diet	Primary antibody X sex	Primary antibody X diet	Sex X diet	Primary antibody X sex X diet
MRA	TH	<0.0001	0.2777	0.6349	0.4268	0.0399	0.2902	0.1479
Intestine	TH	<0.0001	0.6108	0.6922	0.6013	0.7011	0.3807	0.3740
PVAT	TH	<0.0001	0.6467	0.6010	0.7910	0.7085	0.2436	0.1850
Intestine	CD-31	<0.0001	0.5854	0.7726	0.6279	0.8217	0.5921	0.6767
PVAT	CD-31	<0.0001	0.1468	0.0565	0.4442	0.1258	0.1472	0.3514

**FIGURE 13:**
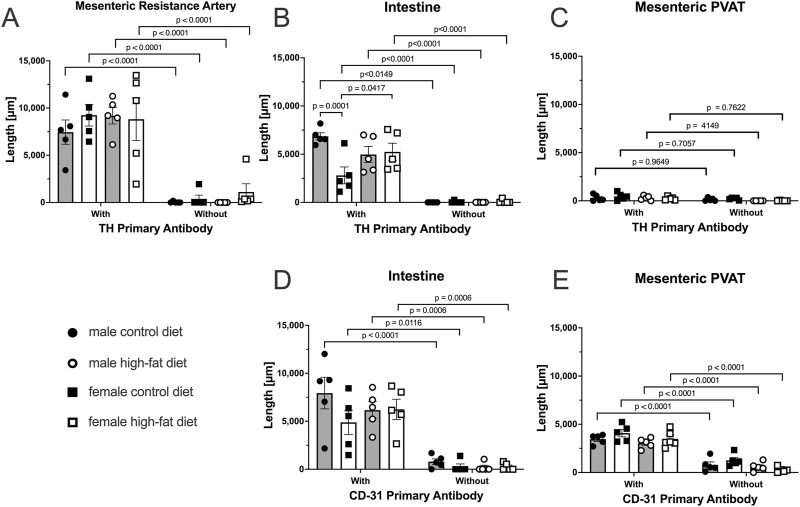
TH-positive nerves are also sparsely distributed in Dahl S mesenteric PVAT when analyzed as total length by NeuronJ. Data are mean ± SE (n = 5) length (μm) of labeled structures for TH (Panels **(A–C)**) and CD-31 (Panels **(D–E)**). The legend shown in the bottom left Panel applies to all other panels in the figure. As in Figure 11, data were statistically analyzed by 3-way ANOVA with primary antibody (with or without), sex (male or female) and diet (control or high fat) as the main treatments with exact p-values shown in Table 2. The p-values shown in the figure refer to comparisons between means using Śídák’s multiple comparison test applied after significant (p < 0.05) ANOVA results. The group variances for the data shown in Panels A and B were found to be significantly different by the Brown-Forsythe test (p-value for Panel A = 0.0292 and for Panel B = 0.0080). Application of a square-root transformation eliminated this issue (p-value after transformation for Panel A = 0.3812 and for Panel B = 0.0818). The p-values shown in Panels A and B were computed on the square-root transformed data.

In reviewing the thresholded images from these ethanol-fixed tissues, greater speckly background staining was noted that likely overestimated the TH-positive structures in all tissues, but especially in PVAT where TH-positive nerves were sparse. We therefore reanalyzed the images for Figure 12 using the NeuronJ plugin for ImageJ (Figure 13). We observed qualitatively similar results after reanalysis of the images. Positive controls (Figure 13A, mesenteric resistance arteries and Figure 13B, small intestine) demonstrated significant TH-labeling similar to that shown in Figures 12A,B. In contrast, there was a very low level of TH-positive nerve-like structures in mesenteric resistance artery PVAT that was not statistically greater than the fluorescence observed in secondary-antibody-only negative controls (Figure 13C). Similar to the analysis shown in Figures 12D,E, CD-31 labeling of small intestine (Figure 13D) and PVAT (Figure 13E) was always observed. The 3-Way ANOVA detected a significant effect of diet on TH labeling in PVAT (p = 0.0473). However, subsequent pair-wise comparisons of means failed to detect significant differences (p > 0.4148 for all comparisons–see Figure 13E for exact p-values). The 3-way ANOVA of TH-staining in intestinal tissue showed, in addition to a significant antibody effect (p < 0.0001), significant sex effect (p = 0.0253) and significant Antibody X Sex (p = 0.0073), Sex X Diet (p = 0.0125) and Antibody X Sex X Diet (p = 0.0129) 2 and 3-way interactions. Subsequent comparisons of means revealed the expected difference between with and without antibodies (p < 0.0001 for all groups). TH-staining was less in females compared to males on control diet (p = 0.0002) and a significant increase in intestinal TH staining in the high-fat fed females compared to control-fed females was observed (p = 0.0338).

**TABLE 2:** 3-way ANOVA p-values for Figure 13.

Tissue	Antibody target	Primary antibody	Sex	Diet	Primary antibody X sex	Primary antibody X diet	Sex X diet	Primary antibody X sex X diet
MRA	TH	<0.0001	0.1127	0.4079	0.2415	0.6790	0.8772	0.1229
Intestine	TH	<0.0001	0.4700	0.0755	0.0073	0.6565	0.0125	0.0129
PVAT	TH	<0.0001	0.0473	0.4562	0.6801	0.9794	0.3079	0.6234
Intestine	CD-31	<0.0001	0.6906	0.1860	0.9464	0.3301	0.1531	0.3198
PVAT	CD-31	<0.0001	0.0113	0.1321	0.8189	0.2670	0.2290	0.4517

## 4 Discussion

Consistent with our report in mouse PVAT (Hanscom et al., 2024), TH-labeling of sympathetic nerves was minimal in rat mesenteric PVAT, with most TH-positive nerves being associated with blood vessels and very few TH-positive nerves appearing to associate with the adipocytes themselves. These findings suggest that PVAT adipocytes that are immediately adjacent to sympathetically innervated blood vessels will be exposed to neurotransmitters like norepinephrine that are released from these nerves. However, because adipocytes take up and store catecholamines (Ayala-Lopez et al., 2014; Ayala-Lopez et al., 2015; Ahmad et al., 2019), adipocytes that are distant from the blood vessels may not be exposed to this neurotransmitter. Our findings suggest that most mesenteric PVAT adipocytes in mice and rats may not be under direct control by sympathetic nerves, as we have previously postulated (Hanscom et al., 2024).

### 4.1 Effects of high fat feeding on adipocyte dimensions and body weight

It is well established that white adipocytes undergo hypertrophy and hyperplasia in obesity (Sun et al., 2011). Consistent with these concepts, high fat feeding led to significant hypertrophy of mesenteric resistance artery PVAT adipocytes in male Sprague-Dawley rats and to a lesser extent in male Dahl-SS rats, but not in female Dahl-SS rats fed the same high-fat diet. In mesenteric resistance artery PVAT from male Sprague-Dawley rats, there was also a significant increase in the width of the PVAT “stripe” around these resistance arteries which we interpret as an increase in adiposity. The high-fat-feeding-induced adipocyte hypertrophy has implications for oxygenation, delivery of metabolic substrates and removal of waste products as the increased cell size will reduce capillary density (as we showed in Sprague-Dawley rat PVAT Figure 5) and increase diffusion distances from capillaries to the surrounding adipocytes as has previously been shown (Paavonsalo et al., 2020).

High fat feeding had no significant effect on body weight in this cohort of Dahl-SS rats after both 10 and 16 weeks on diet (Figure 8D), although male Dahl-SS rats were heavier than females at both 10 and 16 weeks on either control or high Fat diet as previously reported (Alula et al., 2019; Watts et al., 2021). A non-significant weight gain in male Dahl-SS rats was shown previously (Watts et al., 2021). In contrast, female Dahl-SS rats (Watts et al., 2021), both sexes (Alula et al., 2019) and male Dahl-SS rats (Nagae et al., 2009; Beyer et al., 2012) have been reported to gain more weight on a high fat diet than same sex Dahl-SS rats fed a control diet with only 10%–12% fat. We do not know the cause of the differences in diet-induced weight gain in the present (Figure 8D) vs. prior studies (Alula et al., 2019; Watts et al., 2021; Nagae et al., 2009; Beyer et al., 2012). Earlier studies (Alula et al., 2019; Watts et al., 2021) used high fat diets containing only 45% fats vs. the 60% fat that we used here, whereas the same diets were used in the present study and by (Alula et al., 2019; Watts et al., 2021). Thus, it does not appear that differences in diet composition can explain the lack of higher weight gain in Dahl-SS rats on a high fat diet that we observed.

### 4.2 Limited TH-positive nerves in rat mesenteric PVAT

Our findings of limited sympathetic innervation support several prior studies of mesenteric and other white adipose tissue innervation (Ballard and Rosell, 1969; Wirsen, 1964; Slavin and Ballard, 1978; Zeng et al., 2015; Chi et al., 2018). However, they are at odds with studies of the sympathetic innervation of murine inguinal adipose tissue where most adipocytes (91.3% of 30,000) were adjacent to TH-positive nerve fibers (Jiang et al., 2017). The authors of this latter study argue that their method of volume imaging in cleared adipose tissue is simply superior to other methods that have been employed. However, it is more likely that there are regional differences in white adipose tissue innervation as has been reported by others (Ballard and Rosell, 1969; Chi et al., 2018).

### 4.3 Functional studies support heterogeneity in sympathetic innervation in adipose tissue

Functional studies also support the idea that there are regional differences in sympathetic innervation of adipose tissues. Electrical stimulation of nerves significantly increases lipolysis in rat and rabbit epidydimal adipose tissue (Correll, 1963), canine subcutaneous adipose tissue (Rosell, 1966; Fredholm and Rosell, 1968) and canine omental adipose tissue (Ballard and Rosell, 1971). These studies support the hypothesis that there is significant innervation of white adipose tissue adipocytes and control of their function. In contrast, electrical stimulation of nerves in canine mesenteric PVAT have failed to increase lipolysis (Ballard and Rosell, 1971). Furthermore, electrical field stimulation of murine mesenteric PVAT to activate nerves stimulates intracellular Ca^2+^ transients in only 5%–18% of PVAT adipocytes, whereas superfusion of the same tissue with exogenous norepinephrine results in Ca^2+^ transients in >90% of mesenteric PVAT adipocytes (Hanscom et al., 2024). These data support the hypothesis that there are regional differences in adipose tissue innervation that impact the function of PVAT adipocytes.

In contrast to the studies outlined in the last paragraph, electrical field stimulation of mesenteric PVAT released adipokines that inhibit α-adrenergic receptor-induced contraction of rat mesenteric resistance arteries (Bussey et al., 2018; Saxton et al., 2018). The adipokine-release was inhibited by tetrodotoxin (to block nerve action potentials), 6-hydroxydopamine (to deplete catecholamine stores) and β_3_-adrenergic receptor blockade (to block actions of neurally-released catecholamines) (Bussey et al., 2018; Saxton et al., 2018) suggesting a role for sympathetic nerves in this process. Saxton et al. (2018) suggested a dense network of sympathetic nerves in PVAT were responsible for stimulating the release of the adipokines. However, these data are difficult to reconcile with the small effect of electrical field stimulation on adipocyte Ca^2+^ signals in murine mesenteric PVAT and the low density of TH-positive nerves that we and Hanscom et al. (2024) report. One possible explanation to reconcile these data is that only a few adipocytes need to be stimulated to produce a biological response, or that there are non-neural mechanisms of communication among PVAT adipocytes that are involved in the control of PVAT function such as release of anticontractile substances. This could be paracrine chemical communication as it is well known that adipocytes release substances that affect the function of adjacent adipocytes (Saxton et al., 2019a), or that there are cell-cell communication pathways among adipocytes that have yet to be adequately explored. Adipocytes express the gap junction protein, connexin 43, and have been shown to be both dye and electrically coupled (Burke et al., 2014). Thus, limited innervation by sympathetic nerves could still affect a larger area of tissue through cell-cell communication (Zhu et al., 2016). Further research will be required to resolve these issues.

The present study in rat mesenteric PVAT and our recent study in mouse mesenteric PVAT (Hanscom et al., 2024) presenting limited innervation of mesenteric PVAT are difficult to reconcile with the findings of Saxton et al. (2018) who demonstrated dopamine-β-hydroxylase immunostaining around all adipocytes in sections of murine mesenteric PVAT. We simply never saw evidence for this degree of innervation in rat mesenteric PVAT or in murine mesenteric PVAT when labeling for TH. We can only speculate that methodological differences may explain the divergent results that we [present study and (Hanscom et al., 2024)] found compared to those reported by Saxton et al. (2018).

The sparse distribution of TH-positive nerves that we have observed in rat mesenteric PVAT is unlikely due to a technical artifact. We readily detected TH-positive nerves on the surface of mesenteric resistance arteries (Figures 2, 4, 5, 7, 11–13) and within whole-mounts of small intestine (Figures 11–13). Importantly, we clearly detected TH-positive nerves around small blood vessels that course through PVAT usually underneath a layer of adipocytes (Figures 5, 6). Finally, we were able to label endothelial cells in PVAT microvessels and in the intestine using anti-CD-31 antibodies (Figures 4, 10, 12, 13). These data suggest that the low density of TH-positive nerves in mesenteric PVAT may not be simply explained by a lack of antibody penetration or fluorescence visibility.

### 4.4 TH and CD-31 labeling were always observed in positive control tissues

Labeling of TH on the surface of mesenteric resistance arteries and within whole mounts of small intestine support our ability to label TH-containing nerves using the methods described in this study. The pattern of TH-labeling on the surface of mesenteric resistance arteries is similar to previous reports using histochemistry for catecholamines (Furness, 1971) or immunofluorescent staining for TH (Alula et al., 2019). We found a lack of effect of high fat diet on TH-labeling on the surface of mesenteric resistance arteries in both male and female Dahl-SS rats (Figures 12A, 13A). These data are consistent with TH-labeling of mesenteric resistance arteries in control and high-fat-fed Dahl-SS rats previously reported (Alula et al., 2019).

The pattern of labeling of TH that we observed in small intestine is similar to that reported in the literature (see Figure 1 in (Phillips et al., 2013) for example). We did find that TH-labeling in the small intestine of female Dahl-SS rats appeared lower than in small intestine from male rats and that high-fat feeding increased TH-labeling in samples from female rats (Figure 13). The effects of high fat diet on TH-labeling in small intestine have not been previously reported. Additional research will be required to verify these findings and to establish the mechanisms responsible for these differences in TH-labeling in small intestine from female rats.

We were consistently able to label microvessels in PVAT whole-mounts with antibodies directed against CD-31 (Figures 4C,D,I,J, 10E–H,M–P). These data are consistent with the well-established rich vascular supply of white adipose tissue (Ballard, 1978; Rosell and Belfrage, 1979; Crandall et al., 1997; Pasarica et al., 2009; AlZaim et al., 2023; Rauchenwald et al., 2025). In Sprague-Dawley rats where high fat feeding resulted in a near doubling of adipocyte diameter, we observed a reduction of vascular density that is consistent with the adipose tissue capillary rarefaction that has been reported in obesity (Paavonsalo et al., 2020; AlZaim et al., 2023; Ioannidou et al., 2022). We found no effect of high-fat feeding on vascular density in tissues from male Dahl-SS rats (Figures 12E, 13E) despite the small but significant increase in adipocyte size in these animals (Figure 9E) where a proportional decrease in capillary density would be expected. We also found no significant difference in vessel density between male and female Dahl-SS rats (Figures 12E, 13E) despite adipocytes being smaller in the females (Figure 9E). Perhaps the small difference in adipocyte size observed in Dahl-SS rats were simply not large enough to result in detectable changes in vascular density by the methods used in this study. Additional research will be required to address this issue.

Vascular density in the small intestine was similar in control and high-fat fed Dahl-SS and in male and female rats (Figures 11I–L,U–X). This does not appear to have been previously studied, although functional capillary density (capillaries with flowing red blood cells) has been reported using side-stream dark field imaging (Turek et al., 2008). Turek et al. (2008) reported a value of 290 ± 8 cm/cm^2^ (95% CI, n = 5) in male Wistar rats. Given that our images were 425 × 425 μm, and taking the average measured total vessel length equal to 6308.5 ± 2491 μm (95% CI, n = 5 rats), the capillary density per unit area that we measured in the small intestine was 0.0349 ± 0.01378 μm/μm^2^ or 349 ± 137.8 cm/cm^2^ a value not significantly different from the value reported by Turek et al. (2008) (p = 0.3034 by Welch’s t-test).

### 4.5 Limitations

The sample sizes used in these studies were small (3-4 animals/group for Sprague-Dawley rats and 5 animals/group for Dahl-SS rats). To ensure sample representation we collected image stacks from ∼6 random sites in each PVAT sample which had the lowest effect size (defined here as the difference in staining density between samples exposed to primary and secondary antibodies and those exposed to secondary antibody alone) we then used the average of the staining density values as representative for the animal from which the sample was taken. Nonetheless, the statistical power to detect a signal above background for TH-staining in PVAT was likely lower than ideal (define as >80%). However, the effect sizes for TH staining on mesenteric resistance arteries and for TH-and CD-31 staining in small intestine were sufficiently large that statistical power was not limited for those measurements. As they were secondary objectives, we did not power the studies to detect small effects of diet and sex. Nonetheless, we think that our findings suggest that there are no large effects of diet or sex on the labeling that we report in this study.

High-fat feeding did not significantly increase body weight of the Dahl-SS rats in this cohort of rats. This was surprising as prior studies have shown a significant effect of high-fat-feeding on body weight in this strain of rats with a similar time on diet (Alula et al., 2019; Beyer et al., 2012). Nonetheless, this lack of excessive weight gain may explain the lack of effect of high-fat diet on the parameters measured in this study.

### 4.6 Conclusions

In conclusion, we found that there is a low density of TH-positive sympathetic nerves in mesenteric resistance artery PVAT, with most nerves associated with small arteries and arterioles that feed the PVAT microcirculation. These findings suggest that the adipocytes adjacent to these innervated blood vessels will be exposed to sympathetic nerve released neurotransmitters, with most adipocytes being controlled by other mechanisms that remain to be established.

## Data Availability

The original contributions presented in the study are included in the article/supplementary material, further inquiries can be directed to the corresponding author.
